# Prenatal diagnosis of Silver–Russell syndrome with 8q12 deletion including the *PLAG1* gene: a case report and review

**DOI:** 10.3389/fgene.2024.1387649

**Published:** 2024-05-17

**Authors:** Ke Wu, Yuying Zhu, Qiumin Zhu

**Affiliations:** ^1^ Laboratory of Prenatal Diagnosis Center, Quzhou Maternal and Child Health Care Hospital, Quzhou, Zhejiang, China; ^2^ Prenatal Diagnosis Center, Quzhou Maternal and Child Health Care Hospital, Quzhou, Zhejiang, China; ^3^ Obstetrics Department, Quzhou Maternal and Child Health Care Hospital, Quzhou, Zhejiang, China

**Keywords:** Silver–Russell syndrome, obstetrics department, *PLAG1*, loss-of-function, chromosome 8q12

## Abstract

Silver–Russell syndrome (SRS) is a clinically and genetically heterogeneous disorder. A retrospective analysis predicted that the live birth prevalence of SRS in Estonia is 1:15,886 [Yakoreva et al., Eur J Hum Genet, 2019, 27(11), 1649–1658]. The most common causative genetic mechanism in the proband is loss of paternal methylation in the imprinted control region 1 (ICR1) at 11p15.5 chromosome. A few studies suggested that inherited or *de novo* loss-of-function alterations of the *PLAG1* gene, including the whole-gene deletion and intragenic pathogenic variants, could cause a rare type of SRS. To date, less than 20 unrelated *PLAG1*-related SRS cases have been reported, and the clinical information about these cases is limited. We report the first prenatal case of SRS with 8q12 deletion (including the *PLAG1* gene). The fetus presented with intrauterine growth retardation, small for gestational age, relative macrocephaly at birth, and a protruding forehead. Unlike classical SRS cases, the fetus had micrognathia and did not show body asymmetry. We hope that the literature review in this study provides new insights into genotype–phenotype relationships of *PLAG1*-related SRS.

## Introduction

SRS is a rare and well-recognized condition presenting with intrauterine growth retardation (IUGR), small for gestational age (SGA), postnatal growth failure, relative macrocephaly at birth, protruding forehead, body asymmetry, and feeding difficulties and/or low body mass index (BMI). The first international consensus diagnostic clinical criteria and management for SRS were published in 2016 (Wakeling et al., 2017). SRS is a genetically heterogeneous disorder, caused by paternal hypomethylation at chromosome 11p15.5 (SRS1, MIM#180860), maternal uniparental disomy for chromosome 7 (SRS2, MIM#618905), paternally inherited heterozygous *IGF2* variants (SRS3, MIM#616489), heterozygous *PLAG1* variants (SRS4, MIM#618907), and heterozygous *HMGA2* variants (SRS5, MIM#618908). *PLAG1* gene-related SRS4 is quite rarely reported. Since Schinzel et al. (1994) first reported the 8q12 microdeletion-related SRS-like conditions in a 10-year-old girl and suggested certain gene variants or deletions in 8q12 may cause the clinical presentation of SRS. There are only nine documents related to SRS4 for now, including one document related to the prenatal diagnosis of SRS4. In this study, we reported a fetus with a *de novo PLAG1* deletion affected with SRS4. We conducted a literature review to summarize previously reported prenatal/postnatal phenotypes and *PLAG1* genotypes.

## Materials and methods

### Clinical features

At 23 weeks of gestation, a fetal ultrasound scan showed biparietal diameter (BPD = 55 mm) on the 13th centile, abdominal circumference (AC = 182 mm) on the 20th centile, head circumference (HC = 219 mm) on the 68th centile, and femur length (FL = 36 mm) below the 1^st^ centile. Due to abnormal fetal biometry and oligohydramnios, the pregnant woman was referred to the prenatal diagnosis center. The couple did not have a consanguineous relationship, and their family history was not notable. Amniocentesis was performed at 25 weeks of gestation. Trio-based whole-exome sequencing (trio-WES) was recommended. The study was approved by the Medical Ethics Committee of Quzhou Maternal and Child Health Care Hospital (approval KY-2023-11), and written informed consent was obtained from the pregnant woman.

## Methods

### Whole-exome sequencing (WES)

Trio-WES was performed on the genomic DNA (gDNA) of the amniotic fluid sample and peripheral blood from the parents. The xGen™ Exome Research Panel v2 (designed by Integrated DNA Technologies) was used for WES. Quality control (QC) of the DNA library was performed using an Agilent 2100 Bioanalyzer system. DNA nanoball (DNB) preps of clinical samples were sequenced on an ultra-high-throughput DNBSEQ-T7 platform (MGI, Shenzhen, China) with the 150-nt paired-end strategy, following the manufacturer’s protocol. The trimmed reads were then mapped to the UCSC GRCh38 reference genome using Burrows-–Wheeler aligner (BWA) software. The alignment process was refined by local realignment and base-quality-score recalibration steps by means of the Genome Analysis Toolkit (GATK v4.2 software). CNVs were analyzed using CANoe, Convex, XHMM, and ExomeDepth software applications.

### Copy-number variant (CNV) validation

All CNVs identified by trio-WES were verified by real-time quantitative PCR (qPCR), using a novel Duplex TaqMan CNV assay (Applied Biosystems, TaqMan CN early access program; TaqMan probe sequences are available on request). The assays used 20 ng of genomic DNA in a 20 μL reaction. The cycling conditions were as follows: 95°C for 10 min for initial denaturation and enzyme activation, followed by 40 cycles each of 95°C for 15 s and 60°C for 1 min. Relative quantification (RQ) was performed using CopyCaller Software (Applied Biosystems, United States), following the comparative ΔΔCT method.

## Results

### Genetic analysis and confirmation

**FIGURE 1:**
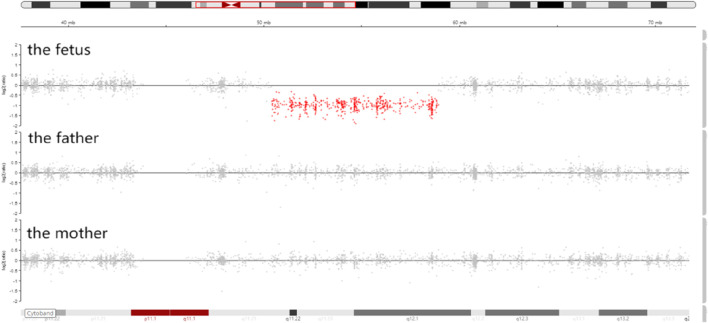
Integrative genomics viewer (IGV) showed that the sequencing coverage of 8q11.21q12.1 in the fetus decreased significantly (the red dots in 8q11.21q12.1 were below the zero line).

**FIGURE 2:**
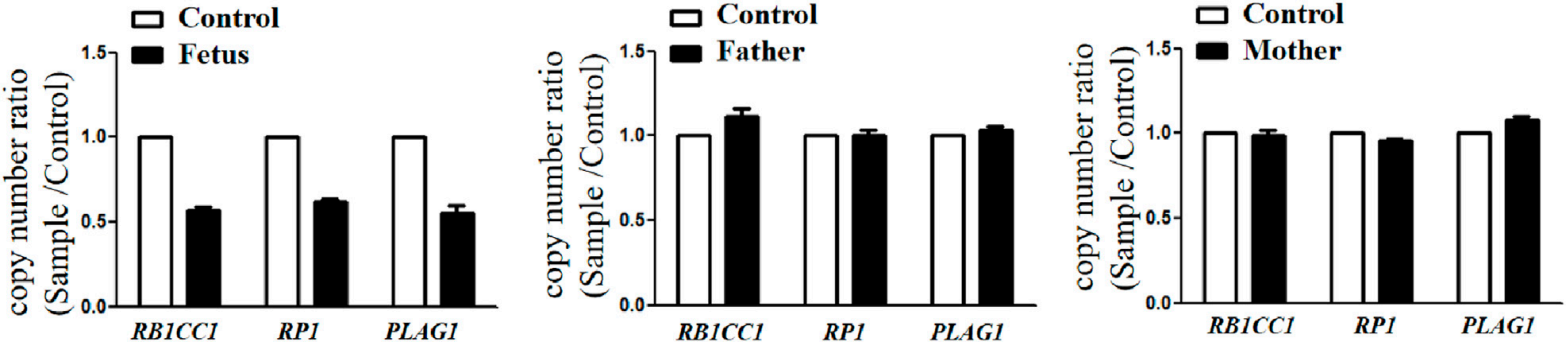
CNVs validation by qPCR showed that the copy number ratio (*RB1CC1*, *RP1*, and *PLAG1* located in the 8q12 deletion) of the fetus was equivalent to the half that of the parents.

Trio-WES identified a *de novo* deletion of 8q11.21q12.1: Seq[hg38]del(8) (q11.21q12.1) chr8:g.50402844_58939544del (Figure 1) including 32 protein-coding genes (*RB1CC1*, *RP1*, *PLAG1*, etc.). All detected CNVs were compared with known CNVs in the scientific literature and with those in the following publicly available databases: Database of Genomic Variants (DGV), DatabasE of genomiC varIation and Phenotype in Humans using Ensembl Resources (DECIPHER), and Online Mendelian Inheritance in Man (OMIM). According to the joint consensus recommendation of the American College of Medical Genetics and Genomics (ACMG) and the Clinical Genome Resource (ClinGen) for reporting constitutional copy-number variants (Riggs et al., 2020), this CNV was a variant of uncertain significance (VUS) (3B: the number of protein-coding RefSeq genes wholly or partially included in the CNV region is 32; the score was +0.45). The CNV validation of the qPCR assay confirmed the result of trio-WES (Figure 2).

### Pregnancy outcome

**FIGURE 3:**
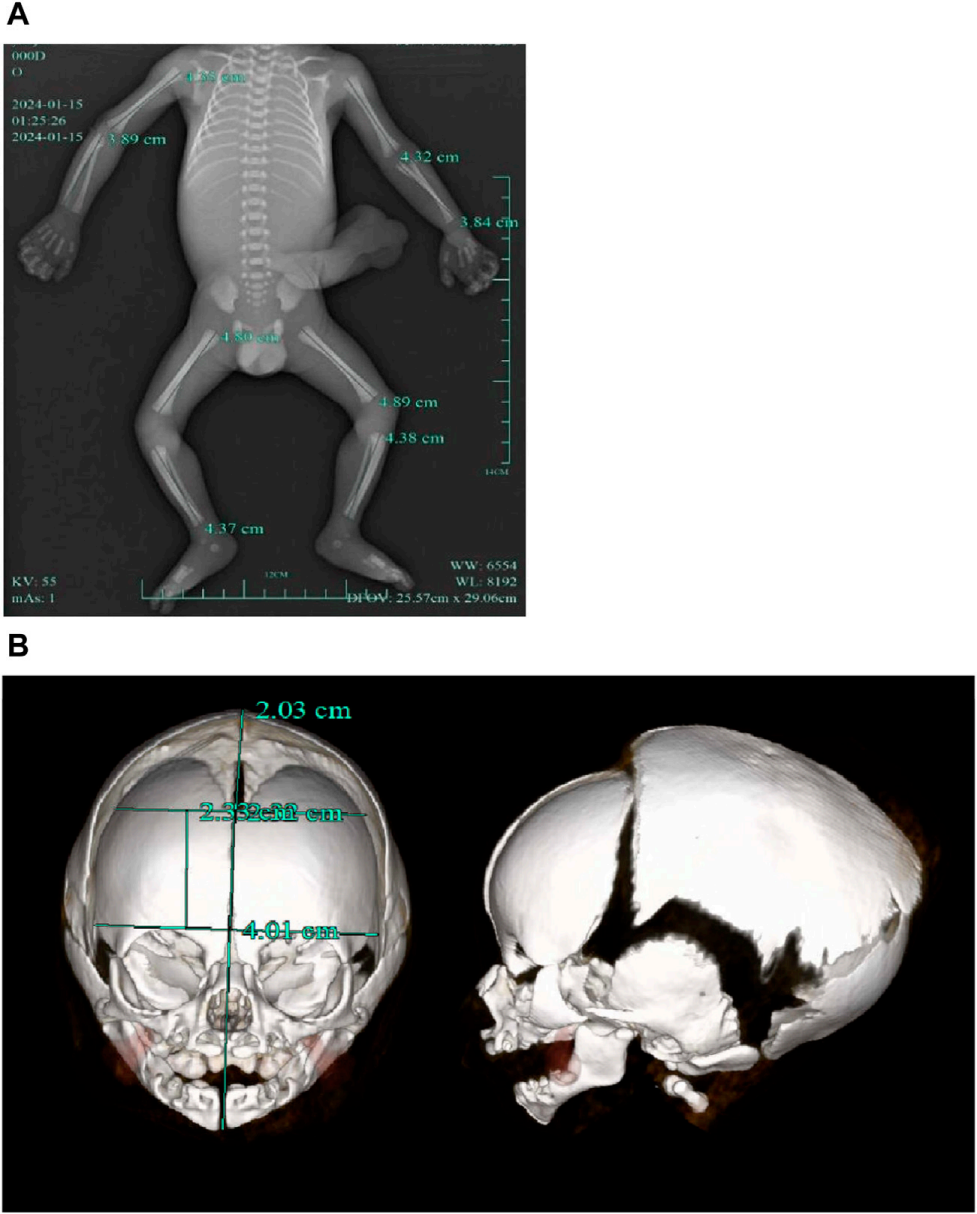
**(A)** CT scan of trunk and extremities did not show body asymmetry. **(B)** The head CT scan showed relative macrocephaly and micrognathia.

**FIGURE 4:**
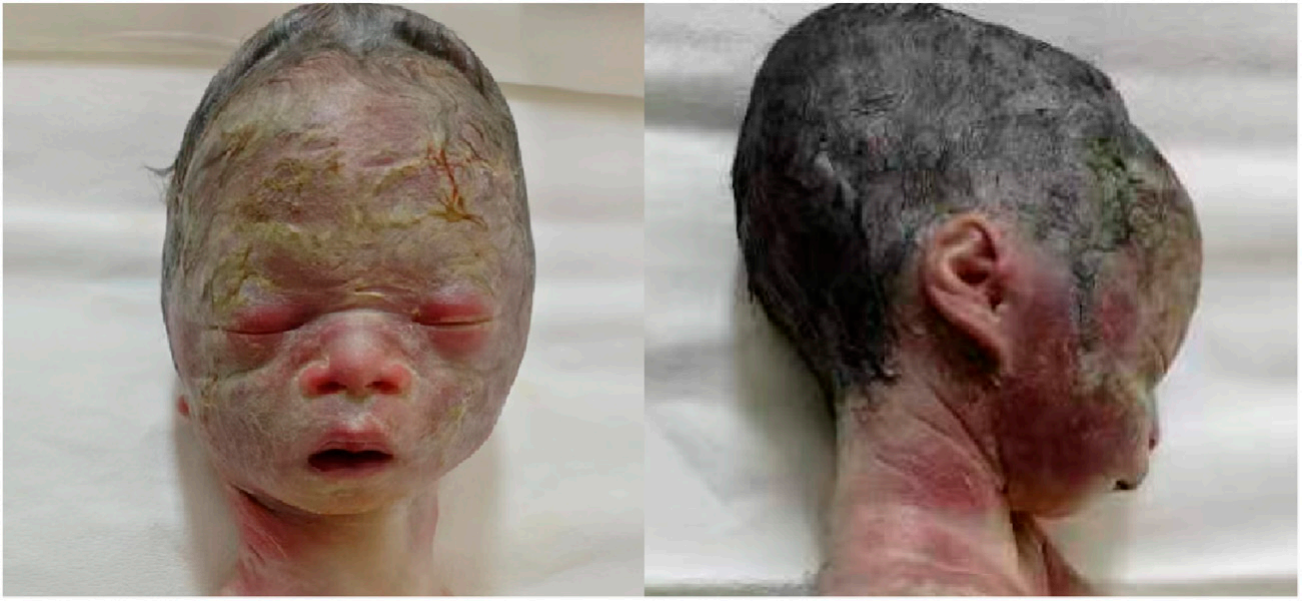
Fetal distinctive facial dysmorphism: prominent forehead, triangular face, orbital hypertelorism, micrognathia, long philtrum, and flat nasal bridge.

A follow‐up fetal ultrasound scan at 29 weeks of gestation showed that fetal biometric parameters including BPD, AC, and FL, were all below the 1^st^ centile except for HC on the 5th centile. The pregnant woman was offered a termination for medical reasons because the genetic tests and fetal ultrasound scan showed that the fetus was not developing as expected. Written informed consent for the fetal computed tomography (CT) scan (Figure 3) and magnetic resonance imaging (MRI) was obtained from the pregnant woman. The CT scan showed that the fetus presented with relative macrocephaly and micrognathia; the fetal trunk and extremities were symmetrical. The MRI of the fetal brain was abnormal. The fetus had a distinctive facial dysmorphism (Figure 4).

## Literature review

We searched the PubMed database, the Human Gene Variant Database (HGMD), and Online Mendelian Inheritance in Man (OMIM) using “Silver–Russell syndrome” and “*PLAG1*” as keywords. The search time was from the establishment of the databases to 31 January 2024. Previous studies with *PLAG1* aberrations and clinical manifestations were reviewed. Ten documents were retrieved. Fourteen postnatal cases with the *PLAG1* gene deletion or intragenic variants and one prenatal case with a heterozygous *PLAG1* gene variant have been reported (Table 1).

**TABLE 1:** Prenatal and postnatal clinical manifestations with *PLAG1* gene aberrations.

Reference	*PLAG1* aberrations (NM_002655.3)	Case number	Age of case report	Gender	Prenatal manifestation	Main postnatal manifestation
Schinzel et al. (1994)	8q11q13 deletion including the *PLAG1* gene	1	9 years old	Female	Diminished fetal activity	Born at 36 weeks of gestation, small for gestational age, microcephaly, prominent forehead, triangular face, fifth finger clinodactyly, muscular hypotrophy, mild delayed motor/speech/language/cognition development, and no body asymmetry
Abi Habib et al. (2018)	c.439del (p.Ser147Valfs*82) (familial cases)	2	NA	Female	NA	Born at term, small for gestational age, prominent forehead, triangular face, and no body asymmetry
		3	NA	Female	NA	Born at 39 weeks of gestation, small for gestational age, no relative macrocephaly at birth, no prominent forehead, triangular face, no body asymmetry, and feeding difficulties during infancy
		4	NA	Female	NA	Born at 39 weeks of gestation, small for gestational age, no relative macrocephaly at birth, prominent forehead, triangular face, feeding difficulties during infancy, and no body asymmetry
	c.1363del (p.Gln455Serfs*16)	5	NA	Female	NA	Born at 31 weeks of gestation, small for gestational age, relative macrocephaly at birth, prominent forehead, triangular face, feeding difficulties during infancy, and no body asymmetry
Inoue et al. (2020)	c.589C>T (p.Arg197*)	6	12 years old	Female	NA	Born at 38 weeks of gestation, small for gestational age, relative macrocephaly at birth, prominent forehead, triangular face, feeding difficulties during infancy, fifth finger clinodactyly, no body asymmetry, no delayed motor/speech/language/cognition development, ADHD, and hypothyroidism
Vado et al. (2020)	c.551del (p.Lys184Serfs*45)	7	9 years old	Female	IUGR	Born at 38 weeks of gestation, small for gestational age, triangular face, feeding difficulties during infancy, no body asymmetry, and delayed motor/speech/language/cognition development
Meyer et al. (2021)	c.599dup (p.Arg201Profs*52)	8	1.5 years old	Female	NA	Born at 36 weeks of gestation, small for gestational age, relative macrocephaly at birth, prominent forehead, triangular face, micrognathia, and no body asymmetry
Brereton et al. (2021)	8q12.1 deletion including the *PLAG1* gene duo to complex chromosomal rearrangements	9	7 years old	Male	IUGR	Born at 38 weeks of gestation, small for gestational age, relative macrocephaly at birth, prominent forehead, triangular face, feeding difficulties during infancy, no body asymmetry, clinodactyly, and neonatal hypoglycemia
		10	2 years old	Male	IUGR	Born at 36 weeks of gestation, small for gestational age, relative macrocephaly at birth, prominent forehead, triangular face, feeding difficulties during infancy, no body asymmetry, delay speech and language development, bilateral clinodactyly, microcolon, and sigmoid atresia
Fernández-Fructuoso et al. (2021)	8q12.1 deletion including the *PLAG1* gene	11	2 years old	Female	IUGR	Born at 32 weeks of gestation, small for gestational age, relative macrocephaly at birth, prominent forehead, triangular face, no body asymmetry, feeding difficulties during infancy, delayed motor/speech/language/cognition development, and micrognathia
Baba et al. (2022)	8q12.1 deletion including the *PLAG1* gene	12	1.5 years old	Male	IUGR	Born at 30 weeks of gestation, small for gestational age, prominent forehead, triangular face, feeding difficulties during infancy, no body asymmetry, and microcephaly
Dong et al. (2023)	c.131del (p.Asn44Thrfs*6)	13	2 years old	Female	NA	Born at term, small for gestational age, relative macrocephaly at birth, prominent forehead, triangular face, no feeding difficulties, no body asymmetry, and no delayed motor/cognition development
Tse et al. (2023)	c.402del (p.Gly135Aspfs*94) (familial cases)	14	25 years old	Female	IUGR	Born at term, small for gestational age, feeding difficulties during infancy, delayed motor development, dextrocardia, atrioventricular septal defect, autism, and none of the physical features of SRS
		15	37 weeks of gestation	Female	IUGR	Born at 37 weeks of gestation and feeding difficulties during infancy
This case	8q12.1 deletion including the *PLAG1* gene	16	29 weeks of gestation	Male	IUGR and oligohydramnios	Relative macrocephaly, prominent forehead, triangular face, micrognathia, and no body asymmetry

Abbreviation: NA, information not available; IUGR, intrauterine growth restriction; SRS, Silver–Russell syndrome; ADHD, attention-deficit hyperactivity disorder.

## Discussion

The *PLAG1* gene, also known as “*pleiomorphic adenoma gene 1*,” is located on chromosome 8q21. The transcript of *PLAG1* (NM_002655.3) has 5 exons, with the transcript length of 7,311 base pairs and translation length of 500 amino acids. It encodes a zinc finger protein, which is broadly expressed during fetal development (Karim et al., 2011), and plays a critical role in the oncogenic HMGA2–PLAG1–IGF2 pathway (Sélénou et al., 2022). PLAG1 acts as a transcription factor whose activation results in the upregulation of target genes (such as *IGF2*), leading to cell proliferation (Hensen et al., 2002). HMGA2 positively regulates IGF2 expression in a PLAG1-independent manner (Abi Habib et al., 2018). IGF2 acts as fetal growth hormone and plays a key role in possessing growth-promoting activity (Cedano Prieto et al., 2017). The loss-of-function (LoF) variants in any of these three genes in this pathway can cause a reduction in IGF2 expression and lead to SRS (Hübner et al., 2020).

To date, there are only 10 documents reporting *PLAG1* gene aberrations associated with SRS4 [14 postnatal cases (case 1–14) and 1 prenatal case (case 15)]. The ratio of male to female patients was 1:4. Differing from classical SRS, all these 16 cases (including our case) did not present with body asymmetry. The most common manifestations (16/16, 100%) were small for gestational age and growth restriction. Other more common manifestations described in documents were triangular face (14/16, 87.5%), prominent forehead (12/16, 68.8%), relative macrocephaly at birth (8/16, 50.0%), and feeding difficulties during infancy (12/14, 85.7%). Some cases showed clinodactyly of the fifth finger (4/16, 25.0%), delayed development of motor/speech/language/cognition (4/14, 28.6%), and micrognathia (3/16, 18.8%). The findings of the three cases (case 1, 12, and 14) least consistent with SRS were their microcephalies and none of the physical features of SRS4. Some uncommon manifestations described in documents were heart defects, microcolon, sigmoid atresia, muscular hypotrophy, hypoglycemia, and autism. The most frequent prenatal manifestation (8/16, 50%) of SRS4 was IUGR. Other nonspecific manifestations were diminished fetal activity and oligohydramnios (our case). The deleted interval wholly or partially involves 32 protein-coding RefSeq genes. Among them, autosomal dominant genes were *PLAG1*, *RP1* (associated with retinitis pigmentosa), *LYN* (associated with autoinflammatory disease), and *SOX17* (associated with vesicoureteral reflux). However, the above mentioned three gene (*RP1*, *LYN*, and *SOX17*)-related phenotypes have not been reported in previous cases with the 8q12 deletion. We speculate that the *PLAG1* gene may be the key haploinsufficient gene on the 8q12 deletion. Three types of *PLAG1* gene variants have been reported: 8q21 deletion (including *PLAG1*) (6 cases), frameshift mutation (9 cases), and nonsense mutation (one case). The most common manifestations in patients with the *PLAG1* deletion (6/6, 100%) were growth restriction, prominent forehead, and triangular face, which were not 100% frequency of occurrence in patients with other *PLAG1* variants. Other more common manifestations described in patients with the *PLAG1* deletion were relative macrocephaly (4/6, 66.7%), feeding difficulties during infancy (4/6, 66.7%), and clinodactyly (3/6, 50%). Differing from other types of *PLAG1* variants, microcephaly only happened to patients with the *PLAG1* deletion. Only one case (1/10, 10%, case 6) with other types of *PLAG1* variants had fifth finger clinodactyly. The frequency of feeding difficulties during infancy in patients with other types of *PLAG1* variants was 70% (7/10), which was no different from patients with the *PLAG1* deletion.

One common pathogenic mechanism of these variations is haploinsufficiency. The probability of the LoF intolerance (pLI) score of the *PLAG1* gene is 1.0, suggesting extremely LoF intolerance (pLI≥0.9). Frameshift and nonsense mutations can cause premature termination codons (PTCs), and then, the aberrant mRNA will be degraded by a nonsense-mediated decay (NMD) pathway, resulting in a complete loss-of-function of the truncated protein (Miller and Pearce, 2014). Although the Clinical Genome Resource (ClinGen) has not yet published curations for the *PLAG1* gene, functional analysis *in vitro* has shown that the whole *PLAG1* gene deletion could result in a reduction in IGF2 (Abi Habib et al., 2018). Recent studies found that deletion mutations within the *PLAG1* gene in goats or cattle were associated with the regulation of growth traits (Wei et al., 2021), (Xu et al., 2018). The manifestations of *PLAG1*-knockout mice suggested that *PLAG1* genes played an indispensable role in postnatal growth and fertility (Juma et al., 2016).

To sum up, we suggest that some clinical manifestations of *PLAG1*-related SRS4 may vary with types of *PLAG1* gene aberrations. Microcephaly and clinodactyly tend to occur in patients with the *PLAG1* deletion. The frequency of growth restriction, prominent forehead, triangular face, and relative macrocephaly is lower in patients with other types of *PLAG1* aberrations. Most (15/16) *PLAG1*-related SRS4 patients presented with the classical clinical manifestations of SRS (apart from body asymmetry). Although we consider *PLAG1* to be a haploinsufficiency gene, further evidence is still requisite.

## Data Availability

The datasets for this article are not publicly available due to concerns regarding participant/patient anonymity. Requests to access the datasets should be directed to the corresponding author.
